# Autocatalytic Sets Arising in a Combinatorial Model of Chemical Evolution

**DOI:** 10.3390/life12111703

**Published:** 2022-10-26

**Authors:** Wim Hordijk, Mike Steel, Stuart Kauffman

**Affiliations:** 1 SmartAnalytiX, 1170 Vienna, Austria; 2 Biomathematics Research Centre, University of Canterbury, Christchurch 8041, New Zealand; 3 Institute for Systems Biology, Seattle, WA 98109, USA

**Keywords:** origin of life, chemical evolution, RAF sets, theory of the adjacent possible

## Abstract

The idea that chemical evolution led to the origin of life is not new, but still leaves open the question of how exactly it could have led to a coherent and self-reproducing collective of molecules. One possible answer to this question was proposed in the form of the emergence of an autocatalytic set: a collection of molecules that mutually catalyze each other’s formation and that is self-sustaining given some basic “food” source. Building on previous work, here we investigate in more detail when and how autocatalytic sets can arise in a simple model of chemical evolution based on the idea of combinatorial innovation with random catalysis assignments. We derive theoretical results, and compare them with computer simulations. These results could suggest a possible step towards the (or an) origin of life.

## 1. Introduction

The idea that chemical evolution led to the origin of life was proposed independently by Oparin [1] and Haldane [2]. It was later shown to be plausible through the experiments of Miller [3]. Recently, different variants of these experiments were repeated, and analyzed with state-of-the-art molecular analysis technology [4,5]. This revealed the presence of thousands of molecular species, including many classes of organic and catalytic ones. However, it still leaves open the question of how such spontaneous chemical evolution can give rise to a coherent, self-reproducing collective of molecules.

One possible answer to this question was proposed in the form of the emergence of autocatalytic sets [6,7]. Informally, an autocatalytic set is a chemical reaction network in which the molecules mutually catalyze each other’s formation, and which is self-sustaining given a basic food source. That such autocatalytic sets can indeed form spontaneously was already shown early on through computer simulations [8,9,10]. Later on, they were also successfully constructed with real molecules in the lab [11,12,13,14], and shown to exist in the metabolic networks of prokaryotes [15,16,17]. The notion of autocatalytic sets was formalized and studied extensively as reflexively autocatalytic and food-generated (RAF) sets (see, for example, [18,19] and references therein).

Recently, a simple model of combinatorial innovation, referred to as the *theory of the adjacent possible* (TAP), was studied formally [20]. In general, TAP states that evolving systems create their own future possibilities in an ever-increasing “adjacent possible”. In particular, the number of “things” that can come into existence (or be created) next increases as a combinatorial function of what is currently in existence. As such, this model can also be interpreted in the context of chemical evolution, where the “things” are molecular species. The more molecular species that are currently in existence, the more potential new species that can come into existence next through (spontaneous) chemical reactions between arbitrary combinations of currently existing molecules. An initial investigation of the combination of the TAP and RAF models was presented in the context of technological evolution [21]. Here, we present results of a further investigation into this model combination, with both theoretical and computer simulation results, and more specifically in the context of chemical evolution and how it could lead to the formation of mutually catalytic and self-reproducing collectives of molecules as a possible step towards the (or an) origin of life.

## 2. Methods

### 2.1. TAP

The TAP model is based on the idea of combinatorial innovation [20]. At its core is the following equation from [22]:

(1) Mt+1=Mt+∑i=1MtαiMti,

where $M_{t}$ is the number of different “things” at time *t*, and $\alpha_{i}$ is a decreasing sequence of probabilities (i.e., real numbers between 0.0 and 1.0). Interpreted in the context of chemical evolution, new molecular species are produced through chemical reactions with arbitrary combinations of already existing species as reactants. More specifically, at each time step *t*, each possible combination of *i* existing molecular species has a small probability $\alpha_{i}$ of chemically reacting, producing a new species. In other words, the probabilities $\alpha_{i}$ could be considered some sort of reaction propensities.

Note, though, that the above equation represents a deterministic version that does not guarantee $M_{t}$ to be an integer value, and it only serves to convey the general idea behind the model. Instead, we use a stochastic implementation that does guarantee integer values, as in [20]. Although our mathematical results do not require it, we will assume for the simulations and the following algorithm that $\alpha_{i}=\alpha^{i}$ (i.e., α to the power *i*).

In addition, for each newly created molecular species *x* and each of the existing molecule types *y* that were not present at time 0, *x* can catalyze the formation of *y* with a fixed probability *p*. Similarly, for each chemical reaction *r* that produces a new species, and each of the existing molecule types *y* that were not present at time 0, *y* can catalyze *r*, also with probability *p*. These random catalysis assignments are assumed to be independent across all pairs of molecules and reactions.

Our implementation of this model is described in Algorithm 1.

Several remarks should be made about this algorithmic description. First, an upper limit *K* on the possible number of reactants is set for numerical and computational reasons. It was already shown earlier that this does not significantly affect the overall behavior of the TAP model [20], and chemically it is a plausible constraint as well.

Next, the algorithm stops when *exactly* M molecular species have been produced (due to the *if*-statement at line 16). Previously, the algorithm was allowed to finish the time step in which M items are reached, in which case the final amount of items is generally larger than M [21]. However, for a more accurate comparison with theoretical results, here we terminate the algorithm immediately after the $M^{\mathrm{th}}$ molecular species has been produced, thus not finishing the rest of the time step in which that happens.

Finally, in the *for*-loop at line 12, a new molecular species *x* is considered twice to catalyze its own production (when $y=M_{t}$). However, the loop is stated this way to keep it more concise, rather than having an additional *if*-statement, or two separate *for*-loops. Of course, in an actual implementation this double consideration can be easily excluded, and as long as $M_{t}$ is large enough, it is negligible in the theoretical derivations below.
**Algorithm 1:** TAP with catalysis.**Require:** $M_{0},\alpha ,K,M,p$1:t←02:Create $M_{0}$ initial molecular species labeled $1,\ldots ,M_{0}$3:**while**$M_{t}<M$**do**4:    t←t+1; $M_{t}\leftarrow M_{t-1}$5:    **for** i=1,…,K **do**6:        si←αi×Mt−1i7:        $r_{i}\leftarrow$ Poisson$\left(s_{i}\right)$8:        **for** $j=1,\ldots ,r_{i}$ **do**9:             Create a new species *x* labeled $M_{t}+1$10:           Select *i* random reactants for the production of *x* from $1,\ldots ,M_{t-1}$11:           $M_{t}\leftarrow M_{t}+1$12:           **for** $y=M_{0}+1,\ldots ,M_{t}$ **do**13:               With probability *p* assign *x* as catalyst to the reaction that produced *y*14:               With probability *p* assign *y* as catalyst to the reaction that produced *x*15:           **end for**16:           **if** $M_{t}=M$ **then**17:               i←K+1; $j\leftarrow r_{i}+1$18:           **end if**19:        **end for**20:    **end for**21:**end while**

**Figure 1:**
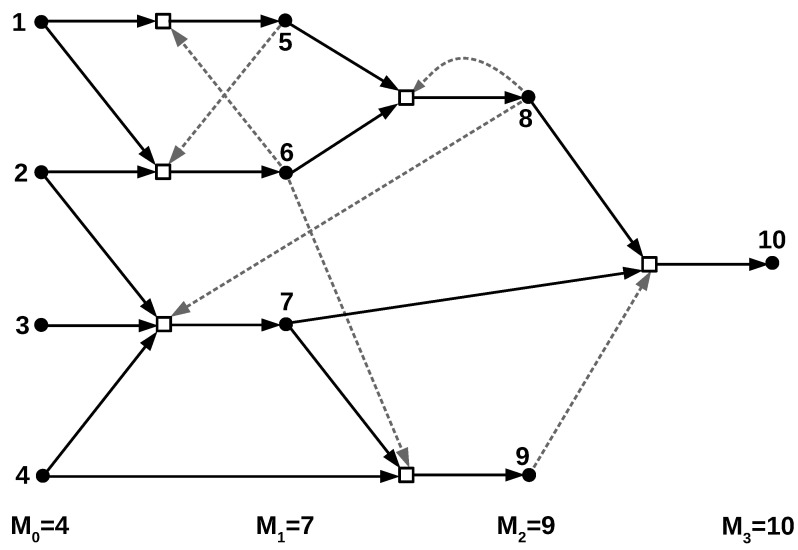
An example chemical reaction network resulting from the TAP model, with random catalysis assignments (dashed arrows). Modified from [21].

Over time, the $M_{t}$ existing molecular species, and the particular chemical reactions that produced them, thus form a growing chemical reaction network, with a “food set” consisting of the $M_{0}$ initial species, as in the example in Figure 1. In addition, the molecules catalyze each other’s formation according to the catalysis probability *p*. Note that each reaction can have no, one, or multiple catalysts, depending on these random catalysis assignments. Similarly, each molecule type may catalyze no, one, or multiple reactions. Figure 1 shows a simple example of a chemical reaction network that resulted from the TAP model, with random catalysis assigned as indicated by the dashed arrows.

As before [21], the parameters $M_{0},\alpha$, and *K* are fixed at the values $M_{0}=10$, α=0.01, and K=4.

### 2.2. RAF

One could now ask what the probability is that in such a growing chemical reaction network, at some point a subset of molecule types exists in which the molecules mutually catalyze each other’s production, and which is sustainable on the given food set. Such a subset is known as a reflexively autocatalytic and food-generated (RAF) set [18]. More formally, an RAF set is a set R of chemical reactions and the molecule types involved in them such that:

1 Each reaction in R is catalyzed by at least one of the molecules involved in R.
2 Each molecule type involved in R can be created from the food set through a sequence of reactions from R itself.

An efficient computer algorithm exists to find such RAF sets in arbitrary chemical reaction networks, or determine that no such subset is present. This algorithm actually finds the (unique) *maximal* RAF (maxRAF), i.e., the union of all possible RAFs within a given network. Repeated application of the algorithm can then also identify smaller RAF subsets within the maxRAF, including minimal ones.

In fact, the entire reaction network in Figure 1 forms a maxRAF, but it contains several smaller RAF subsets (e.g., the two reactions forming the molecule types {5,6} are an RAF, and so too are the four reactions forming {5,6,7,8}).

### 2.3. TAP and RAF

An initial investigation into the existence of RAF sets in the TAP model with catalysis was presented recently, but in the context of technological evolution [21]. Here, we provide a more detailed study, and more specifically in the context of chemical evolution. First, we derive theoretical expressions for the probability of RAFs existing in instances of the TAP model. We then compare these with results from computer simulations, using an implementation of the TAP model as presented in Algorithm 1, and applying the RAF algorithm to large sets of random instances of the TAP model.

## 3. Results

Consider an instance of the TAP model, described by $\left(M_{t},R_{t}\right)$ (for t=0,1,2,…) where $M_{t}$ is a set of molecular species generated up to time *t* and $R_{t}$ is the set of all reactions involved in generating $M_{t}$ starting from $M_{0}$. We let $M_{t}=\left|M_{t}\right|$ and $R_{t}=\left|R_{t}\right|$ denote the number of molecular species in $M_{t}$ and reactions in $R_{t}$, respectively. Note that these families of sets (and their sizes) are random variables for each t≥1. We assume throughout this section that $M_{0}\geq 1$, and $\alpha_{i}\neq 0$ for at least one value of $i\leq M_{0}$. We also explicitly assume in this section that for each pair (x,r) where $x\in M_{t}\setminus M_{0}$ and $r\in R_{t}$, *x* catalyzes *r* with probability *p*, and that these catalysis events are stochastically independent.

Throughout this section we do not specifically require that $\alpha_{i}=\alpha^{i}$, nor do we place any upper bound (such as M) on $M_{t}$ unless otherwise stated, or any bound (such as *K*) on the number of reactants of a reaction.

Since every reaction in the TAP model creates exactly one new product, we have:

(2) $$R_{t}=M_{t}-M_{0}.$$

**Lemma** **1.** *In the TAP model, $M_{t}$ is nondecreasing, and with probability 1, $M_{t}\to \infty$ as t grows.*

**Proof.**  $M_{t}$ is a Markovian random walk on the positive integers, with $M_{t+1}\geq M_{t}$ for all *t*, and the probability of the event $M_{t+1}-M_{t}\geq 1$ is uniformly bounded away from 0 for all values of *t*. By a standard probability argument, for any positive integer *k*, the event $E_{k}$ that $M_{t}\leq k$ holds for all *t* has probability 0. Thus, $P(\cup_{k\geq 1}E_{k})=0$ which ensures that $M_{t}\to \infty$ with probability 1. □

**Remark** **1.** *If an upper bound M is imposed on $M_{t}$ (so that the process terminates when $M_{t}\geq M$) then Lemma 1 implies that $M_{t}$ is certain to eventually hit M. Note also that the version of the TAP model studied theoretically in [20] takes place in continuous (rather than discrete) time, in which case Lemma 1 has a sharper statement: Provided that $\alpha_{1}>0$ and $\alpha_{i}>0$ for at least one other value of i, then with probability 1, $M_{t}$ tends to infinity in* finite *time. Here, we are modeling a system in discrete time, and so this “explosion in finite time” phenomenon does not arise. Nevertheless, in our simulation results, where we explicitly stop the process when M molecular species have been produced, a sudden and rapid increase still occurs.*

Next, consider the probability that the entire collection of reactions involved in generating $M_{t}$ (i.e., $R_{t}$) is an RAF. As the following lemma shows, this probability depends only on $M_{t}$ only through the size of this set (i.e., $M_{t}$), and so we denote the probability by $P_{\mathrm{all}}\left(M_{t}\right)$.

**Lemma** **2.**
(3) $$P_{\mathrm{all}}\left(M_{t}\right)={\left(1-{\left(1-p\right)}^{M_{t}-M_{0}}\right)}^{M_{t}-M_{0}}.$$

**Proof.**  $R_{t}$ is *F*-generated, and so it forms an RAF precisely if each reaction in $R_{t}$ is catalyzed by at least one molecule type in $M_{t}\setminus M_{0}$. By the independence assumption concerning catalysis assignments, the probability that any given reaction $r\in R_{t}$ is catalyzed by at least one molecular species in $M_{t}\setminus M_{0}$ is $1-{\left(1-p\right)}^{M_{t}-M_{0}}$, and since there are $R_{t}$ reactions, the probability that all reactions in $R_{t}$ are catalyzed is ${\left(1-{\left(1-p\right)}^{M_{t}-M_{0}}\right)}^{R_{t}}$ which equals ${\left(1-{\left(1-p\right)}^{M_{t}-M_{0}}\right)}^{M_{t}-M_{0}}$ by Equation (2). □

We can now state our main theorem.

**Theorem** **1.** *For the TAP + catalysis model, the following hold.*

(*a*) *For any value of p>0, $P_{\mathrm{all}}\left(M_{t}\right)=1-o\left(1\right)$, where o(1) is a term that tends to zero as $M_{t}\to \infty$.*

(*b*) *Suppose that $M_{t}=m$ at some fixed time t>0. Then the following hold:*
  (*i*) *If $p=\frac{\ln(xm)}{m}$ for xm>1, then*
$$P_{\mathrm{all}}\left(M_{t}\right)=\exp\left(-\frac{1}{x}\right)+o\left(1\right),$$
    *where o(1) is a term that converges to 0 as m→∞.*
  (*ii*) *The expected number of reactions that each molecule type (not in $M_{0}$) catalyzes (i.e., $f=pR_{t}=p\left(M_{t}-M_{0}\right)$) in order for $P_{\mathrm{all}}\left(M_{t}\right)$ to equal θ is given by:*
$$f=\ln\left(m\right)+\ln\left(\frac{1}{\ln(1/\theta )}\right)+o\left(1\right),$$
    *where o(1)→0 as m→∞. In particular, for each such value of θ, f grows logarithmically with m.*

**Proof.**  *Part (a):* Let $M_{t}^{\prime }=M_{t}-M_{0}$, and let $q={\left(1-p\right)}^{M_{t}^{\prime }}$. By the inequality $1-x\leq e^{-x}$ for x>0, we obtain $q\leq e^{-pM_{t}^{\prime }}$, and thus, by Lemma 2, we obtain:

$$P_{\mathrm{all}}\left(M_{t}\right)={\left(1-q\right)}^{M_{t}^{\prime }}\geq 1-M_{t}^{\prime }q,$$

(since ${\left(1-x\right)}^{n}\geq 1-nx$ for 0<x<1). Thus, $P_{\mathrm{all}}\left(M_{t}^{\prime }\right)\geq 1-M_{t}^{\prime }e^{-pM_{t}^{\prime }}\to 1$ as $M_{t}\to \infty .$

*Part (b-i):* Conditional on $M_{t}=m$, and setting $p=\frac{\ln(xm)}{m}$, Lemma 2 gives:

$$\ln\left(P_{\mathrm{all}}\left(M_{t}\right)\right)=\left(m-M_{0}\right)\cdot \ln\left[1-{\left(1-\frac{\ln(xm)}{m}\right)}^{m-M_{0}}\right]$$

$$∼m\cdot \ln\left(1-e^{-\ln mx}\right)=m\cdot \ln\left(1-\frac{1}{mx}\right)∼-\frac{1}{x},$$

where ∼ refers to asymptotic identity as *m* grows. Exponentiating gives $P_{\mathrm{all}}\left(M_{t}\right)=\exp\left(-\frac{1}{x}\right)+o\left(1\right)$, as required.

*Part (b-ii):* Conditional on $M_{t}=m$, and setting $p=\frac{\ln(xm)}{m}$ for a value x>0 (to be determined), gives:

(4) $$f=pR_{t}=p\left(M_{t}-M_{0}\right)=p\left(m-M_{0}\right)=\ln\left(m\right)+\ln\left(x\right)-pM_{0}.$$

From Part (b), and again conditional on $M_{t}=m$, the equation $P_{\mathrm{all}}\left(M_{t}\right)=\theta$ gives $x=\frac{1}{\ln(1/\theta )}+o\left(1\right)$ where o(1) tends to 0 as *m* grows. Noting also that $pM_{0}=o\left(1\right)$, the result now follows from Equation (4). □

**Figure 2:**
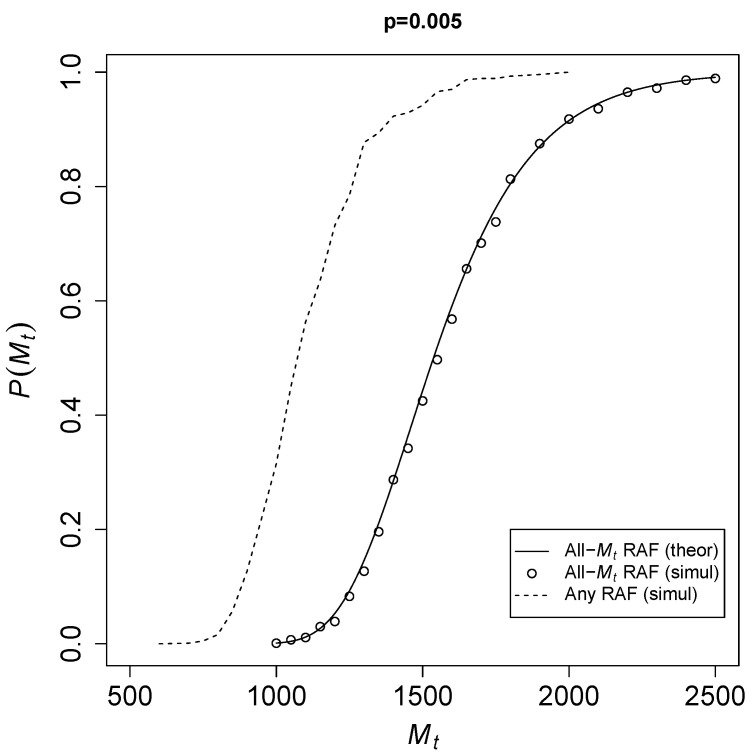
The probability $P(M_{t})$ of RAFs against $M_{t}$ for a catalysis probability p=0.005.

To see how fast $P_{\mathrm{all}}\left(M_{t}\right)$ converges to 1, Figure 2 shows the theoretical probability of Equation (3) for an all-$M_{t}$ RAF (solid line) against $M_{t}$ for a catalysis probability p=0.005. The open circles represent results from the TAP model simulations. The dashed line shows simulation results for any-sized RAF (i.e., an RAF consisting of any number of reactions).

Given a fixed probability of catalysis *p*, it is clear that once the total number of molecular species $M_{t}$ becomes large enough, there is a sharp transition from RAF sets not existing at all to them existing in almost every instance of the model. Of course, RAFs of any size (dotted line) already occur at smaller values of $M_{t}$ than all-$M_{t}$ RAFs, but theoretically it is easier to deal with all-$M_{t}$ RAFs, as they are always automatically food-generated. Thus, the theoretical expression forms an upper bound on the actual probabilities $P(M_{t})$.

**Figure 3:**
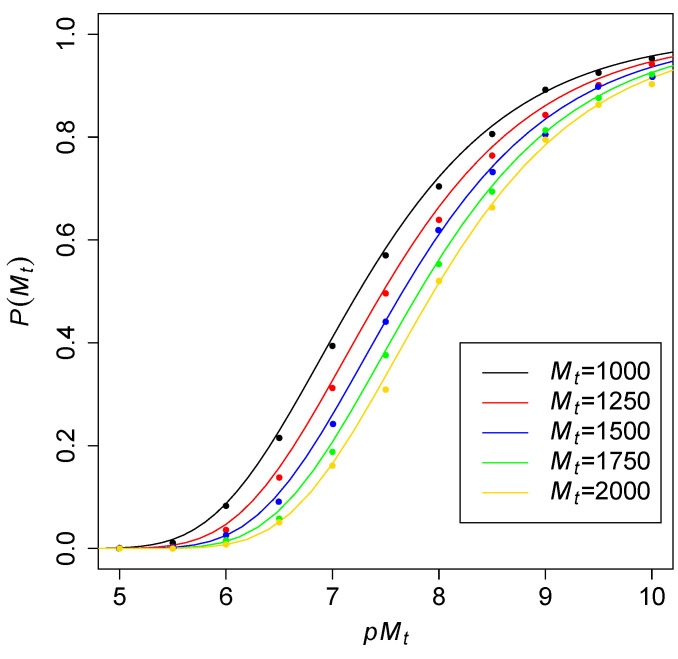
The probability $P(M_{t})$ of all-$M_{t}$ RAFs against $pM_{t}$ for different values of $M_{t}$.

Another way to consider these probabilities is to fix the number of molecular species $M_{t}$ and then see what the required level of catalysis $f=pM_{t}$ is to obtain RAF sets with high probability. This level of catalysis indicates the average number of reactions catalyzed per molecule type. To see how this increases with increasing $M_{t}$, Figure 3 shows the theoretical probability (solid lines) of an all-$M_{t}$ RAF against $pM_{t}$ for different values of $M_{t}$. The dots are values obtained from computer simulations of the TAP model, to again compare with the theoretical results. As expected, the curves move slowly to the right for larger values of $M_{t}$, but the distance between each next pair of adjacent curves seems to be decreasing.

**Figure 4:**
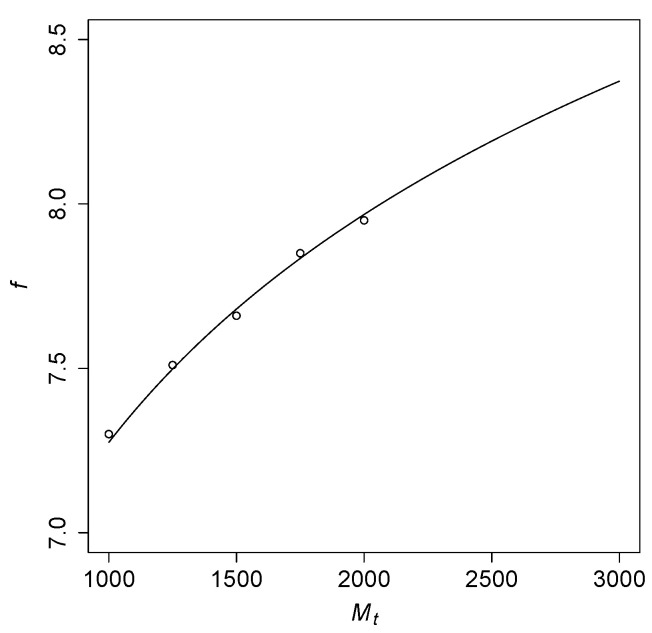
The required level of catalysis $f=pM_{t}$ to obtain all-$M_{t}$ RAFs with a probability 0.5, for a range of $M_{t}$ values.

Taking the “transition point” to a high probability of RAFs to be at $P_{\mathrm{all}}\left(M_{t}\right)=0.5$, Theorem 1(b-ii) predicts that $f=pM_{t}$ should be close to $\ln\left(M_{t}\right)+\ln\left(1/\ln\left(2\right)\right)\approx \ln\left(M_{t}\right)+0.367$. Figure 4 shows this function for a range of values of $M_{t}$, with the open circles representing results from computer simulations (interpolated from the simulation data shown in Figure 3). These simulation results closely fit with the theoretically predicted logarithmic curve.

Finally, a comparison is made between the probabilities of any-sized RAFs previously obtained from simulations of the TAP model [21], and earlier results from a related model known as the binary polymer model [23]. In this related model, smaller polymers can ligate into larger and larger ones, and larger polymers can cleave into smaller and smaller ones. This model has been investigated extensively in the context of RAF sets in the past [18,19].

**Figure 5:**
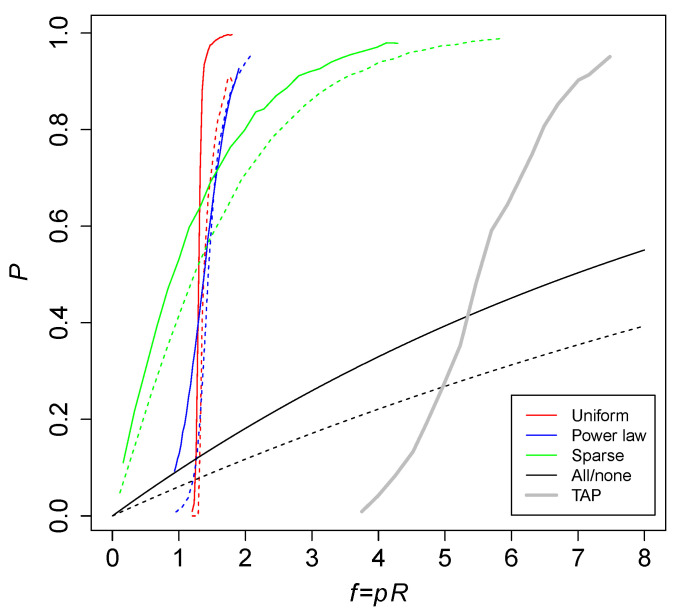
The probability *P* of RAFs in different versions of the binary polymer model (solid lines for n=10 and dashed lines for n=16) and in the TAP model ($M_{t}=1250$). Adapted from Hordijk and Steel [23].

Figure 5 shows the probability *P* of an RAF against the level of catalysis f=pR for various versions of the binary polymer model that use different ways of assigning catalysis. The red curves are the standard (uniform) catalysis distribution, the blue curves are a power law catalysis distribution, and the green lines are a sparse catalysis distribution [23]. These results were obtained from computer simulations. The black curves are the theoretically calculated probabilities for an all-or-nothing catalysis distribution. Solid lines are maximum polymer length n=10, while dashed lines are n=16. The thick gray line shows simulation results for the TAP model, with an average number of molecular species of $M_{t}=1250$.

Clearly, the required level of catalysis to cause RAF sets to arise is higher in the TAP model (five to seven reactions catalyzed per molecule type, on average) than in the binary polymer model (one to two). However, this can be explained by the fact that in the TAP model there is always only one reaction that produces a given molecular species, whereas in the binary polymer model there are multiple reactions that can produce a given polymer. In other words, there is a large amount of redundancy in the reaction networks resulting from the binary polymer model, allowing for a lower level of catalysis (given that only one or two of the multiple reactions that produce a given polymer need to be catalyzed).

Note also that we restricted the chemical reactions in the TAP model to only generate one product. In general, reactions can produce more than one product, in which case there could be significantly more molecular species than reactions. It is therefore expected that in such a more general model version the required level of catalysis *f* will be lower than the five to seven suggested by Figure 5.

## 4. Conclusions

We reinterpreted a simple model of combinatorial innovation known as TAP (theory of the adjacent possible) in the context of chemical evolution and autocatalytic sets. We then derived theoretical expressions for the probabilities of such autocatalytic sets arising in instances of the TAP model. These theoretical predictions were verified with results from computer simulations.

These results show that autocatalytic sets do indeed have a high probability of arising in instances of the TAP model, given a large enough number of molecular species and/or level of catalysis. Of course this is still a very general model, as it is assumed that any molecular species can chemically react with any other, and catalysis is assigned randomly. However, previous work on a related model, known as the binary polymer model, showed that more realistic assumptions can be easily incorporated in such a model, and do not change the overall results very much, at least not qualitatively. Moreover, the quantitative changes can often be predicted from the more general basic model version [18].

Generally, the level of catalysis (i.e., the average number of reactions catalyzed per molecule type) needs to be somewhat higher in the TAP model than in the binary polymer model. However, this can be explained by the redundancy present in reaction networks resulting from the binary polymer model. One could easily imagine a version of the TAP model where certain molecular species can also be produced by multiple reactions.

In conclusion, the results presented here may suggest a possible step towards the (or an) origin of life, where self-sustaining and reproducing autocatalytic sets arise during a process of chemical evolution. In fact, Wollrab et al. [4] conclude from their “Miller-type” chemical evolution experiments that “organic catalysts that appear in the broth may well lead to the production of molecular species that would normally not be favored under the conditions in the reactor, further enhancing the molecular richness”. If even just some of those species happen to form a closed loop, mutually catalyzing each other, autocatalytic sets would indeed arise spontaneously.

## Data Availability

All data was generated with custom-made software. Independent verification of our results would be greatly appreciated.
